# Genetic diversity among reptilian orthoreoviruses isolated from pet snakes and lizards

**DOI:** 10.3389/fvets.2023.1058133

**Published:** 2023-02-02

**Authors:** Renáta Varga-Kugler, Katalin Ihász, Szilvia Marton, Eszter Kaszab, Rachel E. Marschang, Szilvia Farkas, Krisztián Bányai

**Affiliations:** ^1^Veterinary Medical Research Institute, Budapest, Hungary; ^2^National Laboratory for Infectious Animal Diseases, Antimicrobial Resistance, Veterinary Public Health and Food Chain Safety, Budapest, Hungary; ^3^Laboklin GmbH & Co. KG, Bad Kissingen, Germany; ^4^Department of Obstetrics and Food Animal Medicine Clinic, University of Veterinary Medicine, Budapest, Hungary; ^5^Department of Pharmacology and Toxicology, University of Veterinary Medicine, Budapest, Hungary

**Keywords:** *Reptilian orthoreovirus*, phylogeny, next generation sequencing, snake, lizard

## Abstract

Reovirus infections in reptiles are frequently detected and associated with various clinical diseases; yet, our knowledge about their genetic diversity and evolutionary relationships remains limited. In this study, we characterize at the genomic level five reptile origin orthoreovirus strains isolated from exotic snakes and lizards in Hungary and Germany. The genomic organization of the study strains was similar to that of the representative strains of reptile origin reoviruses belonging to species *Reptilian orthoreovirus* and *Testudine orthoreovirus*. Additionally, all five study strains clustered with the bush viper origin reference *Reptilian orthoreovirus* strain, 47/02. The nucleotide sequence divergence among strains fell from 56.64 to 99.36%. Based on genome segment constellations two well separated groups were observed, which may represent two genetic lineages of reptilian orthoreoviruses we tentatively referred here as genogroups, classifying two squamata origin strains with available whole genome sequences into genogroup I (GGI) and four strains into genogroup II (GGII). The representative GGI and GGII *Reptilian orthoreovirus* strains are characterized by moderate-to-high nucleotide and amino acid similarities within genogroups (range, 69.45 to 99.36% and 74.64 to 100.00%), whereas lower nucleotide and amino acid similarities (range, 56.64 to 77.24% and 54.53 to 93.85%) and different structures of the bicistronic S1 segment were found between genogroups. Further studies are needed to explore the genomic diversity among reptilian reoviruses of squamata origin; this would be critical to establish a robust classification system for these viruses and to see if interaction among members of distinct lineages may result in viable progenies with novel genetic features.

## 1. Introduction

Members of the order *Reovirales* are double-stranded RNA viruses infecting a wide range of host species, including plants, fungi, protists and animals. The order is divided into two families, *Sedoreoviridae* and *Spinareoviridae* containing 6 and 9 genera, respectively. According to the International Committee on Taxonomy of Viruses (ICTV) the genus *Orthoreovirus* belonging to the family *Spinareoviridae*, is currently divided into ten species: *Avian orthoreovirus* (ARV), *Baboon orthoreovirus* (BRV), *Broome orthoreovirus* (BroRV), *Mahlapitsi orthoreovirus* (MAHLV), *Mammalian orthoreovirus* (MRV), *Nelson Bay orthoreovirus* (NBV), *Neoavian orthoreovirus* (NeARV), *Piscine orthoreovirus* (PRV), *Reptilian orthoreovirus* (RRV) and *Testudine orthoreovirus* (TRV) (1, 2). Orthoreoviruses are non-enveloped viruses with an icosahedral capsid 70–80 nm in diameter. The 23 kilobasepairs (kbp) viral genome consists of 10 segments grouped into three categories based on their size: three large segments (L1-L3), three medium segments (M1-M3), and four small segments (S1-S4). With the exception of the S1 or S4 segment, which might be bi- or tricistronic, each genome segment encodes a single protein (3).

The genome size of reptile origin orthoreoviruses is about 24 kbp (range, 23,957 to 24,043 bp, based on two fully sequenced viral genomes) (4, 5). Genetic analysis of a short fragment of the RNA-dependent RNA polymerase (RdRp) gene identified two or three genetic clades; however more recent data indicate that reoviruses of reference snake and tortoise origin strains belong to different *Orthoreovirus* species (4–8). At present, the few known isolates of reptilian reoviruses are officially classified into two distinct *Orthoreovirus* species: *Reptilian* and *Testudine orthoreovirus*, latter represented by only a single strain isolated from a spur-thighed tortoise (5, 7). Reovirus infections in reptiles may be asymptomatic, but have been associated with various clinical diseases and experimental infection induced severe respiratory disease in snakes (9, 10). Reptilian orthoreoviruses induce giant cells *in vivo* and *in vitro*, a feature linked to the expression of fusion associated small transmembrane (FAST) protein encoded by the S1 genome segment (6, 10, 11). Although reptilian reoviruses are easily isolated and frequently detected, our knowledge about their genetic diversity and evolutionary relationships remained limited.

Genetic diversity within orthoreoviruses stems from accumulation of point mutations generated by the viral RdRp that lacks proofreading activity and genetic reassortment of cognate genomic segments, which may result in new combinations of gene constellations (12, 13). In addition to reassortment events among members of a particular virus species, exchange of homologous genomic segment has been hypothesized to occur among viruses belonging to different *Orthoreovirus* species (14, 15). Moreover, intrasegmental recombination involving cognate genomic segments may also occur among homologous viruses and this mechanism may further increase the genetic diversity within orthoreoviruses (15).

In the present study we performed whole genome sequencing of five reptilian orthoreovirus strains isolated from different exotic species in Hungary and Germany. Because of the segmented nature of their genome and the evidence of various reoviral strategies to increase genetic diversity, all genome segments were subjected to phylogenetic calculations in order to uncover their evolutionary relationships.

## 2. Materials and methods

### 2.1. Virus detection and isolation

**Figure 1:**
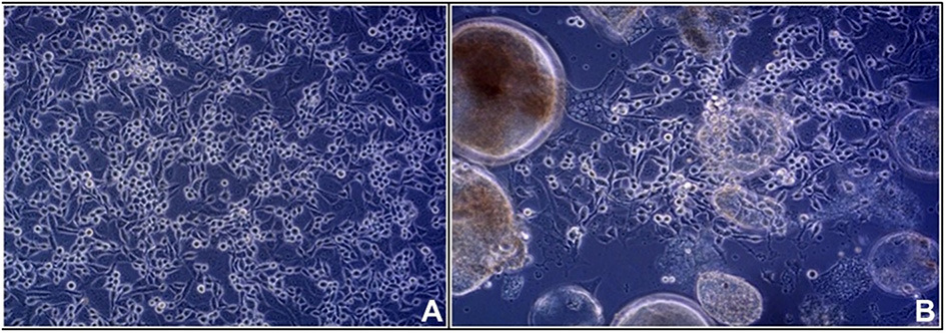
Viper heart (VH2) cell line: **(A)** control **(B)** syncytium formation induced by infection with KP3 reptilian orthoreovirus strain.

The strains analyzed in the current study are listed in Table 1. Strain IBD26/00 was isolated from the liver of a *Boa constictor* that died with inclusion body disease as described elsewhere (16). For strains 2013/12, 2013/54, 2013/47, and KP3, organ samples (heart, tongue, throttle, lung, esophagus, stomach, intestine, liver, and kidney) were collected in sterile PBS solution from succumbed animals. We have no information about the health, body condition and the circumstances of death of the animals. A pan-reovirus-specific reverse transcription nested polymerase chain reaction targeting a conservative region of the orthoreoviral RdRp gene was used for detection of reptilian orthoreoviruses from the supernatant of the pooled organ samples (8). In order to isolate the orthoreoviruses, the supernatants of the pooled organ samples were propagated in a 6 well culture dish on viper heart (VH2) or iguana heart (IgH2) continuous cell lines. After 4 days when cytopathic effect appeared, isolates were used for VH2 or IgH2 cell lines applying 75-cm^2^ flasks. On day 4 post infection when syncytium formation appeared (Figure 1) the virus isolates were harvested by freezing and thawing. Cell culture supernatants containing the virus isolates were stored at −80°C and periodically passaged on VH2 or IgH2 cells to maintain their viability.

**Table 1:** List of virus strains analyzed in the current study.

**Sample**	**Species**		**Isolation**		**Source**
			**Location**	**Year**	
IBD26/00	Boa constrictor	*Boa constrictor*	Germany	2000	Pet owner
KP3	Ball python	*Python regius*	Budapest, Hungary	2013	Pet shop^*^
2013/12	Schneider's skink	*Eumeces schneideri*	Budapest, Hungary	2013	Pet shop^*^
2013/54	Green iguana	*Iguana iguana*	Budapest, Hungary	2013	Pet shop^*^
2013/47	Unknown snake		Hungary	2013	Pet owner

^*^Originated from the same pet shop.

### 2.2. Whole genome sequencing

The viral RNA was extracted from polyethylene glycol concentrated cell culture supernatant using TRIzol Reagent (Sigma-Aldrich) according to the manufacturer's recommendations. Extracted RNA was processed for whole genome sequencing as described in details previously (4). Random primed reverse transcription was used to generate complementary DNA (cDNA) from viral RNA. cDNA libraries were prepared using the NEBNext^®^ Fast DNA Fragmentation & Library Prep Set for Ion Torrent (New England Biolabs, Beverly, MA, USA) using the Ion Torrent Xpress barcode adapters (Life Technologies, Carlsbad, CA, USA) according to the instructions recommended by the manufacturers. The emulsion PCR and subsequent templated bead enrichment were performed with a OneTouch v2 instrument and Ion OneTouchTMES, respectively. Sequencing was carried out on a 316 chip using the Ion Torrent Personal Genome Machine^®^ (Life Technologies). To obtain the 5' and 3' terminal sequences of the segments, DNA oligonucleotides were ligated to each end of the dsRNA as described in detail elsewhere (17, 18). The confirmation of next generation sequencing (NGS) data and the completion of missing parts of the sequences was carried out with additional oligonucleotide primers in PCR and Sanger sequencing reactions (not shown).

### 2.3. Computer analysis

Sequence reads generated by IonTorrent sequencing were assembled using the CLC Genomics Workbench version 7 (http://www.clcbio.com). Contigs were aligned with Sanger sequencing reads and were edited using Geneious Prime and AliView softwares (19, 20). BLASTn and BLASTx algorithms were used to identify homologous genes among sequences deposited in GenBank (21). Codon-based multiple sequence alignments were generated using the Muscle algorithm within the TranslatorX (22). Phylogenetic analysis was performed and sequence identity values were calculated using the MEGA6 and MEGAX package (23, 24). Gene-specific substitution models were evaluated and the best-fit models were selected based on the Bayesian information criterion. Maximum-likelihood trees were generated, and tree topologies were validated by bootstrap analysis (100) as implemented in MEGAX. A short fragment of the RdRp gene was analyzed by neighbor-joining method. In aim to discover potential recombination events Recombination Detection Program v4.100 (RDP4) was used applying the default parameters for the embedded methods BootScan, Chimera, GENECONV, MaxChi, RDP, SiScan, and 3Seq (25).

### 2.4. Sequence data availability

**Figure 2:**
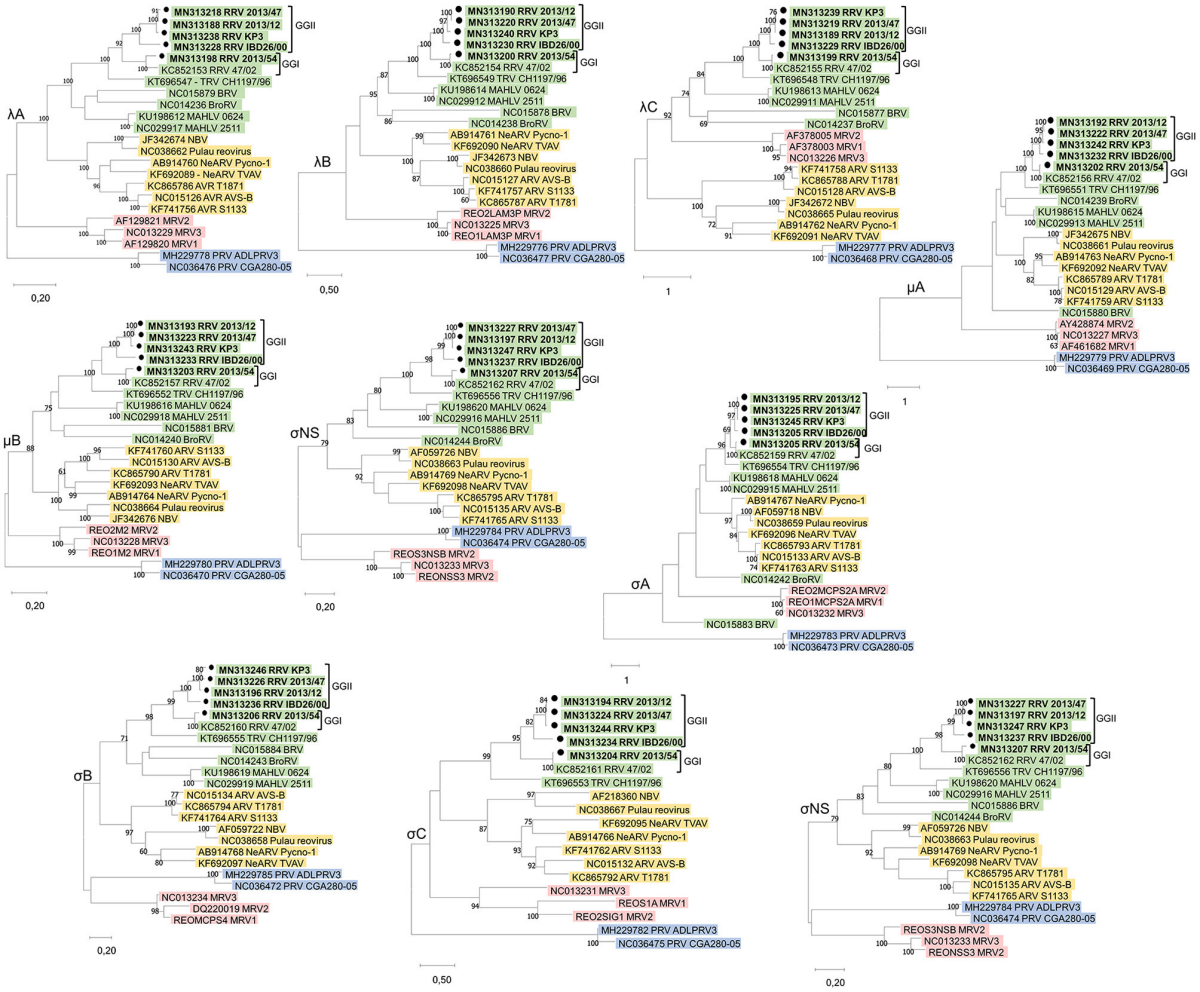
Unrooted phylogenetic trees based on the nucleotide sequences of the corresponding genes of different orthoreoviruses. Phylogenetic trees were calculated by the maximum-likelihood method based on the best-fit model selected for each gene. The scale bar is proportional to the genetic distance. Bootstrap values >60 are shown at the branch nodes. ARV, *Avian orthoreovirus*; BRV, *Baboon orthoreovirus*; BroRV, *Broome reovirus*; MAHLV, *Mahlapitsi orthoreovirus*; MRV, *Mammalian orthoreovirus*; NBV, *Nelson Bay orthoreovirus*; PRV, *Piscine orthoreovirus*; RRV, *Reptilian orthoreovirus* and TRV, *Testudine orthoreovirus*.

The genome sequences of strains 2013/12, 2013/54, 2013/47, IBD26/00, and KP3 were deposited in the GenBank database under the accession numbers MN313188–MN313197, MN313198–MN313207, MN313218–MN313227, MN313228–MN313237, and MN313238–MN313247, respectively. Additional sequence data were obtained from GenBank. The accession numbers of the corresponding segments of the reference reovirus strains are shown on the phylogenetic trees (Figure 2).

## 3. Results and discussion

### 3.1. Genomic features

Five reovirus strains were isolated in 2000 in Germany and around a decade later in Hungary (Table 1). All of the detected viruses possessed the ability to cause giant cell formation in cell culture (Figure 1), similar to other reptilian orthoreoviruses (6, 10, 11). The complete genomes of the strains ranged from 23,990 to 24,033 bp in length and the size of the individual genome segments ranged from 1,209 bp (S4) to 3,970 bp (L1) (Table 2). The G+C content was 45.9–47.6%. The genomic organization of the strains was similar to that of the two previously described reptile origin reovirus strains, 47/02 and CH1197/96 (4, 5). With the exception of S1, which was bicistronic in the studied strains, all genome segments were monocistronic. Using the Geneious Prime software and the BLASTx algorithm the following orthoreoviral proteins were identified by ORF prediction and sequence comparison: λA (core shell), λB (core RdRp), λC (core turret), μA (core NTPase), μB (outer shell), μNS (NS factory), σA (core clamp), σB (outer clamp), σNS (NS RNAb), σC (outer fiber), and p14 (FAST) (4, 26). The 3′ untranslated regions were 41-104 nt long and included the UCAUC 3′ terminal consensus sequence conserved between *Orthoreovirus* species. The 5′ untranslated regions were 12–31 nt long and found to be highly conserved at the termini with the exception of the fourth position which can hold an adenine or a cytosine (Table 2).

**Table 2:** General genomic features of the studied strains.

**Genome segment**	**Size (nt)**	**Length (nt) of**			**Sequence at the termini 5' end/ 3' end**	**Encoded protein**	**Protein size (aa)**
		**5' UTR**	**ORF**	**3' UTR**			
L1	3,967–3,970	13	3,870–3,873	84	GUUCUU/UUCAUC	λA (core shell)	1,289–1,290
L2	3,901–3,933	14	3,846–3,870	41–49	GUUCUU/UUCAUC	λC (core turret)	1,281–1,289
L3	3,847–3,849	14–16	3,786	47	GUUCUU/UUCAUC^#^	λB (core RdRp)	1,261
M1	2,480–2,488	27	2,373–2,382	78–81	GUUAUU/UUCAUC	μNS (NS factory)	790–793
M2	2,339–2,349	13–14	2,232–2,283	51–104	GUUCUU/UUCAUC^*^	μA (core NTPase)	743–760
M3	2,130–2,134	26	2,031–2,034	73–74	GUUAUU/UUCAUC	μB (outer shell)	676–677
S1	1,499–1,503	22–23	360–381	66–70	GUUAUU/UUCAUC	p14 (FAST)	119–126
			1,050–1,053			σC (outer fiber)	349–350
S2	1,314	12	1,251	51	GUUAUU/UUCAUC	σA (core clamp)	416
S3	1,279–1,288	31	1,161–1,170	86–87	GUUAUU/UUCAUC	σB (outer clamp)	386–389
S4	1,209–1,212	24–25	1,110–1,113	74	GUUAUU/UUCAUC	σNS (NS RNAb)	369–370

^#^Except: IDB 26/00 GUUAUU. ^*^Except: 2013/54 GUUAUU.

### 3.2. Comparison with representative members of other *Orthoreovirus* species

Nucleotide (nt) sequences of each genome segment were analyzed separately. With the exception of the ORFs encoding the proteins μA and σA, in which case BRV and BroRV clustered separately, phylogenetic analysis revealed four well separated lineages within the genus *Orthoreovirus*, reaffirming previous findings: (i) viruses representing the three serotypes of the classic MRV (Jones, Lang, Dearing); (ii) ARV and NeARV with those of bat origin: NBV and Pulau reoviruses; (iii) RRV and TRV along with BRV, BrRV, and MAHLV; and (iv) PRV (Figure 2) (4, 27, 28). The sequence identity values of homologous genes and the proteins they encode ranged between 29.86–63.98% and 10.84–66.72%, respectively, when study strains were compared with representative members of the non-reptilian origin *Orthoreovirus* species (data not shown). These identities mostly remained below the cut-off value currently used as demarcation criteria for distinct species within the genus *Orthoreovirus* (< 60% for nt, < 65% for the core protein aa and < 35% for the outer capsid proteins aa sequence), but in the comparison of the μB protein we found higher identities that were above this cut-off value. The μB protein tended to be conserved to a similar degree as the core proteins as observed previously for other orthoreoviruses (5, 29). Furthermore, in the comparison with MAHLV we found identity values above the demarcation criteria defined for distinct species for three additional sequences (λB 65.76–66.72% aa; λA 60.37–62.23% nt; and λB 62.37–63.98% nt identity) confirming the tree topologies where MAHLV appeared to be the closest relative of reptile origin orthoreoviruses (27, 30, 31).

### 3.3. Comparison with reference reptilian and testudines RVs

Orthoreoviruses isolated from reptile hosts presented in this study composed one monophyletic clade together with the two previously described orthoreoviruses from reptiles, one isolated from a bush viper (*Atheris squamigera*) and the other from a spur-thighed tortoise (*Testudo graeca*), suggesting a common evolutionary relationship of these viruses (4, 5). All of the newly described reoviruses originating from different squamata species composed a common group with 47/02, the reference strain of the species *Reptilian orthoreovirus*, which was also isolated from a squamate host. On the other hand, CH1197/96, the reference strain of *Testudine orthoreovirus* clustered separately in all phylogenetic trees indicating differences in orthoreoviruses of phylogenetically distant reptilian hosts (Figure 2).

**Figure 3:**
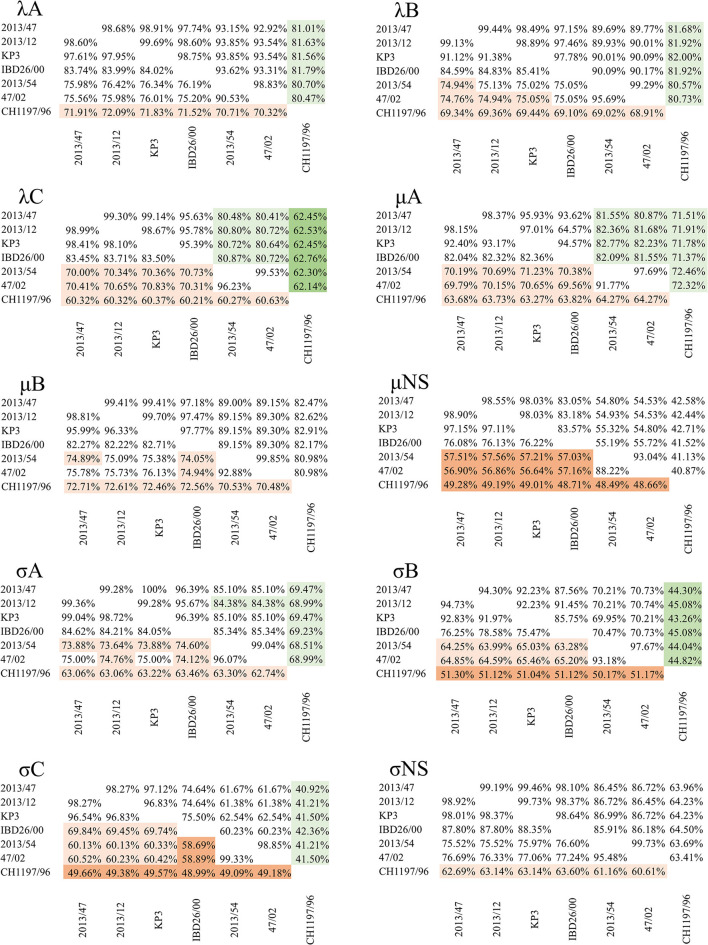
Pairwise identity values of nucleotide **(lower left triangle)** and amino acid **(upper right triangle)** sequences of the corresponding genes from reptilian origin orthoreoviruses. Values under the demarcation criteria for different species are indicated by dark orange (nt) and dark green (aa); values between the two cut-off values (i.e., above the cut-off value for different but under the value for identical species) are indicated by light orange (nt) and light green (aa) colors.

Confirming our previous findings on the existence of different reptile origin reovirus species, comparison of each of the newly described squamate reoviruses with sequences from CH1197/96 showed that none of the identity values reached the cut-off defined for identical species (>75% for nt, >55% for the core protein aa and >85% for the outer capsid proteins aa sequence) (5). Furthermore, identity values remained below the species demarcation criteria for distinct *Orthoreovirus* species in the case of μNS (48.49–49.28%), σB (50.17–51.30%), and σC (48.99–49.66%) nucleotide and λC (62.14–62.76%) protein sequences (Figure 3).

### 3.4. Phylogeny of the genome of squamata reoviruses

Among the newly described reptilian orthoreoviruses two different lineages can be observed: strain 2013/54 (green iguana, *Iguana iguana*) composed a clade with reference RRV strain 47/02 (bush viper, *Atheris squamigera*) while strains 2013/12 (Schneider's skink, *Eumeces schneideri*), 2013/47 (unknown snake species) and KP3 (ball python, *Python regius*) made up another clade together with IBD 26/00 (boa constrictor), the latter clustering on a separate branch but in close relationship with the other two isolates (Figure 2). We clustered these lineages into genogroups (GG) and designated 47/02-like strains as GGI and KP3-like strains as GGII. Most of the sequence identities between the strains within each of the two genogroups reached the demarcation criteria for identical species (Figure 3). The only exception was the σC nucleotide sequence, in which case we found slightly lower values (69.45–69.84%) between IBD26/00 and the three other strains within the same lineage (2013/47, 2013/12, KP3).

In the comparison between the two genogroups, the results were more diverse. In the pairwise comparison of the nucleotide sequence data only the λA (75.20–76.42%) and σNS (75.52–77.24%) nt identities reached the cut-off value defined for identical species. In addition, the identity values for the μNS (56.59–57.56%) and σC (58.50–60.52%) ORFs remained below the cut-off value for distinct species. In the case of the other six genes, the identities fell between the two cut-off values, which gives no recommendation for species classification: λB (74.76–75.13%), λC (70.00–70.83%), μA (69.56–71.23%), μB (74.05–76.13%), σA (73.32–75.00%), and σB (63.28–65.46%).

The identity values calculated for the amino acid sequences of the outer capsid proteins were all above the species demarcation criteria for identical species (μB 89.00–89.30%, σB 69.95–70.74%, and σC 60.23–62.54%) as were the values for the λA (92.92–93.85%) and λB (89.69–90.17%) inner core proteins. However, the analysis of the more conserved inner core proteins revealed somewhat lower amino acid identities, falling under the cut-off value for identical species, in the case of λC (80.48–80.87%), μA (80.87–82.77%), and σA (84.38–85.34%) proteins.

The pairwise identity values affirmed the lineage diversification seen on the phylogenetic trees; strains grouping on different branches showed only moderate or low similarity. On the other hand, strains belonging to the same lineage showed high similarity. Also, the structure of the bicistronic S1 segment was slightly different in the two groups; GGII strains (2013/12, 2013/47, IBD26/00, and KP3) possessed two overlapping ORFs on the S1 segment, while the ORFs on the S1 segment of GGI strains (47/02 and 2013/54) were non-overlapping. Based on RDP4 analysis, recombination event was not detected in the protein coding genes of the squamata origin orthoreoviruses including the reference strain of RRVs and strains analyzed in this study.

## 4. Concluding remarks

Data about the genetic diversity among reptilian orthoreoviruses is scarce and most data obtained so far comes from animals held in captivity. Infection of wild reptiles with orthoreovirus has not been thoroughly investigated and thus the epizootiology of this virus in nature remains largely unclear; nonetheless, the few serological investigations performed to date confirmed the circulation of reoviruses under natural circumstances (32, 33). In this study, we described and analyzed five reptile origin orthoreovirus strains at the genomic level. The study strains originated from snakes and lizards, whose carcasses were obtained from pet owners and pet shops in two European countries, Germany and Hungary. Although the sample size was small and the strains were collected only from two countries within a narrow timeframe, this study enabled the comparison of orthoreoviruses with diverse host species origins. Apparently, the strict regulations on the trade of reptiles and the limited sources of animals may, at some extent, limit the diversity of known virus variants circulating among captive snakes and lizards and the observed viral diversity may not necessarily reflect the true viral diversity which could be isolated from populations of wild reptiles. Despite these shortcomings, our analyses revealed some notable features of the genetic diversity within reptile origin reoviruses.

**Figure 4:**
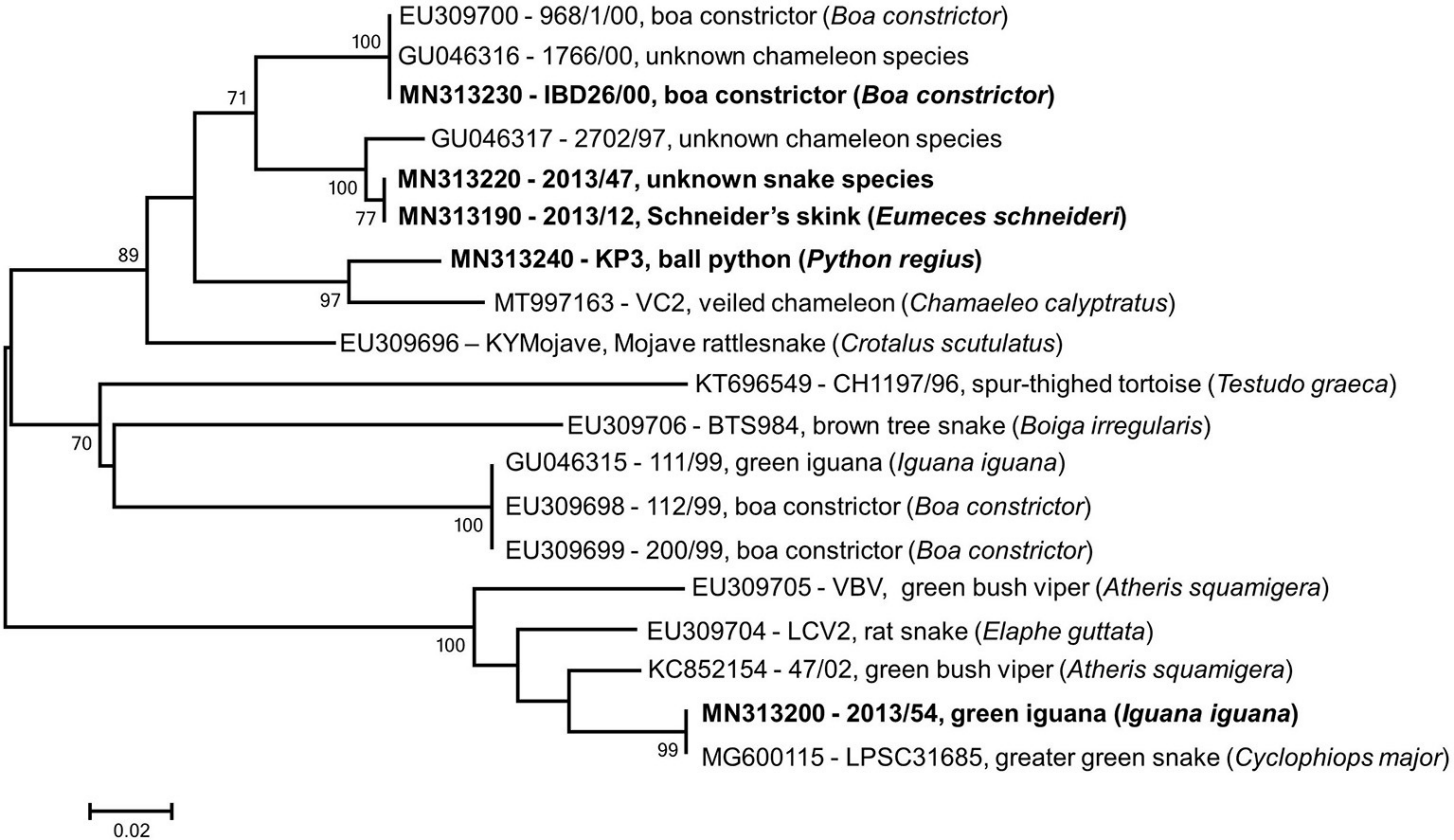
Phylogenetic tree based on partial RdRp gene using overlapping regions of reference strains available in GenBank. Taxa are identified by accession number, strain name and host species; study strains are highlighted. The scale bar is proportional to the genetic distance. Bootstrap values greater than 60 are shown at the branch nodes.

All lizard and snake origin reovirus strains we analyzed in this study comprised a common phylogenetic cluster (*Reptilian orthoreovirus*) and showed the closest relationship with the only representative of the *Testudine orthoreovirus* (4, 5). Using available GenBank entries we generated an alignment containing short overlapping fragments of the RdRp gene (λB); next, we performed phylogenetic analysis that revealed additional diversity among squamata origin orthoreoviruses (Figure 4). In this analysis, three and five reference strains could be classified into the proposed genetic lineages, GGI and GGII, respectively. In addition, another two putative lineages of squamata origin reptilian orthoreoviruses, represented by strains BTS984 and 111/99, respectively, were identified. Although these strains showed low sequence similarity along a ~100 nt long fragment with the single known member of *Testudine orthoreovirus*, they seemed to share common evolutionary roots. In general, evidence shows that very short gene sequences might be suitable to perform taxonomic classification of very diverse viruses (8, 34, 35). However, experience with reptilian reoviruses is limited and further data are needed to determine whether these divergent unclassified reovirus strains are members of the newly established *Testudine orthoreovirus* species, or, they represent distantly related lineages of the *Reptilian orthoreovirus* species, or, they constitute completely new virus species. The extension of genome sequencing will be required to update and validate the current taxonomy of reptilian reoviruses and establish a robust classification system below the virus species level.

## Data availability statement

The data presented in the study are deposited in the GenBank repository under the accession numbers MN313188-MN313197, MN313198-MN313207, MN313218-MN313227, MN313228-MN313237, and MN313238-MN313247.

## Author contributions

KB and SF contributed to the study conception and design. Material preparation, data collection, and analysis were performed by RV-K, KI, SM, EK, and RM. The first draft of the manuscript was written by RV-K and all authors commented on previous versions of the manuscript. All authors read and approved the final manuscript.
